# Report of the Surgical Cases Admitted under the Care of the Surgeon of the Finsbury Dispensary, St. John's Square, Clerkenwell, from the 10th of May, to the 10th of June, 1802

**Published:** 1802-08-01

**Authors:** J. Ricards

**Affiliations:** Surgeon to the Finsbury Dispensary. Hatton-Street


*Report of the Surgical Cases admitted under the Care of the Surgeon of the Finsbury Dispensary, St. John's Square, Clerkenwell, from the 10th of May, to the 10th of June, 1802.*

| | | | |
|---|---|---|---|
| Inflammation of the Extremities | 3 | Fiftula in Ano | 2 |
| ——— of the Breaft | 1 | Ulcers of the lower Extremities | 11 |
| ——— of the Eye | 1 | Wounds of the Extremities | 7 |
| ——— Lippitudo | 1 | ——— of the Head | 1 |
| Abceffes in the Axilla | 1 | Luxation of the Shoulder | 1 |
| ——— in the Groin | 1 | Contufions | 7 |
| ——— of the Knee | 1 | Sprains | 2 |
| ——— Whitloes | 3 | Cancer of the Cheek | 1 |

Lues

| | | | |
|---|---|---|---|
| Lues - - - - - - - - - | 2 | Hemorrhoids - - - - - | 1 |
| Scrophula - - - - - - - | 1 | Paralytic Extremity - - - | 1 |
| White Swellings of the Joints | 2 | Stricture - - - - - - - | 1 |
| Enlarged lymphatic Gland | 1 | Verruca - - - - - - - - | 1 |
| Steatomatous Tumour - - | 1 | Tinea - - - - - - - - - | 1 |
| Varix - - - - - - - - - | 1 | Herpes - - - - - - - - | 1 |

A regular Report of caſes which occur at any public Inſtitution, belonging peculiarly to the Surgeon, is at preſent a novelty in periodical works, thoſe Reports which are now publiſhed being confined principally to the diſeaſes which are with more propriety to be referred to the Medical department. The reaſon of this preference is probably ariſing from the idea that Medical Reports are thoſe alone which can benefit ſociety, as they principally depend on circumſtances of locality, atmoſphere, and various concurring cauſes, which it is not preſumed have any influence on caſes which belong more eſpecially to the Surgeon. If ſuch is the idea, it appears founded on error; for although a great number of ſurgical caſes ariſe from cauſes which may operate at all times and in all ſeaſons, and at any place, yet there are ſtill many which are influenced materially by different circumſtances of locality, and variations in the ſtate of the circumferent agents. A fracture or a diſlocation may occur at any time or place, if the exciting cauſes be applied; and there are various of the diſeaſes which come under the cognizance of the Phyſician, which may happen at any time without a reference to any of the ſurrounding external cauſes. But when a great accident has been inflicted, as its well-doing depends, in a great meaſure, upon the ſtate of the vital energy of the patient, or his general health, ſo it muſt be influenced by every cauſe which influences the general ſyſtem, or accelerates or retards its actions.

There may be reckoned two general circumſtances or diviſions among external cauſes which have a peculiar influence on diſeaſe; theſe are, firſt, the variations which are perpetually occurring in the ſtate of the atmoſphere in which we live; and, ſecond, the particular habitudes or modes of life of the patient. Theſe circumſtances tend in many inſtances to give birth to diſeaſe; and not only ſo, but in various other cauſes which ariſe perhaps from other exciting cauſes, they give the peculiar figure to the diſeaſe, mark out the type which it is to aſſume, give riſe in the courſe of the complaint to peculiar phenomena, which we are from hence taught to account for, and by knowing their cauſe in many inſtances are enabled to obviate, they influence the progreſs and the termination of the diſeaſe.

Theſe two general heads of external cauſes may be divided into numerous parts. Under the firſt may be reckoned the various various changes which take place in the temperature, humidity, &c. of the atmofphere, the directions and fudden alterations in the winds, atmofpherical electricity, &c. &c. Under the fecond head are to be confidered all the caufes arifing from, or referable to, the habits of life of the patient: his fituation with refpect to the neceffary articles of fuftenance, as fome of the clafs of perfons who apply for relief at inftitutions of this kind are in the habits of free living, and very frequently fuffering from intoxication, while others are in want of the moft common and neceffary articles of life; the habitations, cloathing, degree of cleanlinefs, &c. muft all be confidered under this head.

All thefe circumftances not only influence, but give rife to difeafe; and it is not merely in difeafes which fall to the care of the Phyfician that they manifeft their agency, but in thofe which are ufually denominated furgical; as, with refpect to the firft general head, we can trace the effects of a north wind with as much accuracy in a luxated or fractured limb, as in a peripneumony or phthifis. Nothing is more influenced by thefe external caufes from the particular ftate of conftitution which they induce, than the procefs of ulceration; we mark the effects of warmth, and the different changes of the atmofphere; the extremes of poverty; the want of thofe neceffaries which contribute to the continuance and the comforts of life: A ftate of wretchednefs, diet, and debility, are peculiarly characterized in the appearance of the ulcer, diftinguifhing it very forcibly from the fore of the hard-working, hard-drinking, and robuft labourer; both of which claffes of people are not uncommon objects of public benevolence.

Again, the habits of life of the poor, conduce, in a very peculiar manner, to fpecific difeafes, which, affuming different appearances, fall under the care of either the Phyfician or the Surgeon, according to the form of the difeafe; as, the different fpecies of cutaneous affections, which are, many of them, principally arifing from the admiffion of dirt and filth between the fkins, which induce difeafed actions of the parts. The various forms of fcrophula we daily fee invading the lowly habitations of the poorer claffes of fociety, and committing its ravages under the moft dreadful forms: the Phyfician is daily called upon to adminifter to this difeafe, when exifting in the form of phthifis, tabes mefenterica, &c. &c. and the Surgeon has, every day, frefh objects prefented to him, with enlargements of the lymphatic glands, difeafed joints, &c. &c. arifing from this dreadful malady.

I would not wifh here to be underftood to infinuate that fcrophula is confined to humble life; as it is a difeafe depending upon upon delicacy of ſtructure, that ſtate may be, and conſtantly is, produced by various exciting cauſes, which induce a debile frame in all ſtations of life; beſides, as it is frequently an hereditary diſeaſe, and as, in a few generations, the fortunes of life often ſo wonderfully change, we cannot be ſurpriſed that it ſhould invade all claſſes of the community. So intereſting a ſubject ſhall be particularly commented on in a ſubſequent Report.

But it may be urged, that the principal advantage reſulting from periodical Reports, is the detailing of epidemic diſeaſes, which are ſuppoſed peculiarly to belong to the Phyſician: This muſt be granted; yet, notwithſtanding this advantage, the report of Caſes of Surgery, though not perhaps ſo immediately uſeful for this intention, muſt be productive of conſiderable advantage, if properly conducted, as in courſe thereof many obſervations muſt naturally occur, on particular diſeaſes, or general phyſical cauſes, which could never appear by any other channel.

As the preſent Paper is to be conſidered as introductory, it may be proper to ſay a few words on the mode intended to be adopted in making theſe Reports. The uſual mode is by a heterogeneous mixture of Engliſh and Latin names, and ſome of theſe extremely unſcientific. The moſt ſcientific method would be, in reporting Medical Caſes, to follow the order of the beſt Noſologiſt; but as no correct noſology of diſeaſes which may be denominated chirurgical has yet appeared, ſuch a plan cannot be adopted in the preſent inſtance; I ſhall therefore adhere to the following order of arrangement:

1ſt. Idiopathic inflammation, as being a morbid action the moſt general of any, being confined to no particular part, and being produced at different times by ſuch various and different cauſes, and whoſe terminations and effects produce a majority of the Caſes of Surgery.

2d. The various terminations and conſequences of inflammation, as abſceſſes, ulcers, fiſtulæ, &c. &c.

3d. Accidents.

4th. Specific diſeaſes, depending on a particular morbid poiſon.

Laſtly. The various local diſeaſes, not ariſing from any manifeſt external accidental cauſes, but depending on a particular action of the part itſelf, or from internal cauſes.

In the names of the diſeaſes, I ſhall adhere to the general Engliſh appellation, as the publication for which this Report is intended, is not confined to the inſpection of the Medical World, but diffuſed throughout the ſtudies of the public in general; but in thoſe inſtances where a Latin name has, through through uſe, become anglicized, I ſhall prefer it to a tranſlation of the word into Engliſh, which might appear formal and pedantic.

J. RICARDS,
Surgeon to the Finſbury Diſpenſary.

*Hatton-Street.*

